# Effect of infusion direction on convection-enhanced drug delivery to anisotropic tissue

**DOI:** 10.1098/rsif.2024.0378

**Published:** 2024-10-02

**Authors:** Yi Yang, Tian Yuan, Ferdinando Rodriguez y Baena, Daniele Dini, Wenbo Zhan

**Affiliations:** ^1^ School of Engineering, University of Aberdeen, Aberdeen, UK; ^2^ Department of Mechanical Engineering, Imperial College London, London, UK

**Keywords:** convection-enhanced delivery, anisotropy, mathematical modelling, drug transport

## Abstract

Convection-enhanced delivery (CED) can effectively overcome the blood–brain barrier by infusing drugs directly into diseased sites in the brain using a catheter, but its clinical performance still needs to be improved. This is strongly related to the highly anisotropic characteristics of brain white matter, which results in difficulties in controlling drug transport and distribution in space. In this study, the potential to improve the delivery of six drugs by adjusting the placement of the infusion catheter is examined using a mathematical model and accurate numerical simulations that account simultaneously for the interstitial fluid (ISF) flow and drug transport processes in CED. The results demonstrate the ability of this direct infusion to enhance ISF flow and therefore facilitate drug transport. However, this enhancement is highly anisotropic, subject to the orientation of local axon bundles and is limited within a small region close to the infusion site. Drugs respond in different ways to infusion direction: the results of our simulations show that while some drugs are almost insensitive to infusion direction, this strongly affects other compounds in terms of isotropy of drug distribution from the catheter. These findings can serve as a reference for planning treatments using CED.

## 1. Introduction

The results of conventional intravenous drug delivery to treat brain diseases are often disappointing in clinical practice. This is mainly due to the blood–brain barrier (BBB) that blocks over 98% of drugs in the bloodstream [1], leading to a serious lack of drug availability within the lesion for effective therapy. Convection-enhanced delivery (CED) has been developed to overcome this barrier using a catheter to directly infuse drugs into brain tissue [2]. Although the feasibility and safety of this delivery method have been demonstrated in preclinical trials [3], its clinical performance in treating brain disorders including brain cancer and Parkinson’s disease remains low [4]. A major limitation is identified as the difficulty in controlling drug distribution [5].

Drug delivery in the brain is composed of multiple physicochemical and physiological processes that are affected by the intracerebral environment. In particular, the widely distributed axons form numerous cable-like bundles that take on considerably different orientations. This tissue microstructure marks the brain white matter significantly anisotropic and thence influences drug transport and distribution in space [6]. Notably, the drug infusion through a catheter, which can be controlled in practice, is also highly directional. This fact raises the potential to improve drug distribution by adjusting the catheter placement relative to the local axon bundles, which can be precisely controlled in clinical practice. However, the role of infusion direction in convection-enhanced drug delivery to anisotropic tissues is multifactorial and not yet clear.

The tissue environment for drug delivery needs to be maintained consistently when determining the role of influential factors. This requirement is difficult to meet through clinical means due to the complexity and dynamic variations *in vivo*. As an alternative, mathematical modelling has been developed to use a set of governing equations to describe the interconnected drug transport steps, allowing the determination to be conducted in an individual or integrated manner. The pioneering model was initially established in one dimension to study the impact of interstitial pressure and convection on the delivery of antibodies in cancer therapy [7]. The model was later extended to three dimensions to predict drug distribution in space. Particularly, by incorporating the fluid filtration and permeability maps derived from dynamic contrast-enhanced magnetic resonance imaging (MRI) data, the model was applied to reveal the role of heterogeneous vasculature in interstitial transport in a murine sarcoma [8]. Another modelling study compared the transport and accumulation of four types of anticancer drugs delivered through implementable polymeric wafers to a brain tumour, which was rebuilt in three dimensions from patient medical images [9]. The model was also tailored to predict intracerebral drug delivery through pressure-driven infusion. Based on a three-dimensional brain model reconstructed from patient MRI data, the modelling study thoroughly analysed multiple factors impacting drug delivery to brain tumours through CED [10]. These factors included tumour hydraulic conductivity, pore size on the vasculature wall and catheter misplacement outside the tumour. The performance of CED infusion to the intact brain tumour and remnant brain tumour post-operation was also examined for different drugs numerically [11,12]. However, most simulations assumed brain tissue to be isotropic due to the lack of realistic tissue properties. A significant improvement was the development of an algorithm to extract tissue anisotropy from patient diffusion-tensor imaging (DTI) data [13,14]. Using data from MRI and positron-emission tomography (PET), patient-specific analyses were carried out to compare the modelling predictions with experimentally measured CED delivery outcomes [15]. This study identified the potential causes of errors in modelling studies and the essential role of infusion-induced expansion and loss through a compromised BBB in CED treatment [15]. In another representative study, an algorithm developed based on the drug delivery model and DTI proved to have high prediction accuracy and great promise for treatment planning after being validated with experimental data measured *in vivo* [16]. A parametrical study was also performed to determine the effect of tissue permeability and drug diffusion anisotropy on drug distribution in CED treatments [17]. Nonetheless, the placement of infusion catheters is often idealized. The effect of infusion direction on drug delivery through CED to anisotropic tissues is still unclear.

In this study, a mathematical model is applied to a three-dimensional configuration to determine the impact of infusion direction on the delivery of six different drugs to anisotropic tissues through CED. The key processes involved in convection-enhanced drug delivery are considered, including interstitial fluid (ISF) flow, drug convective and diffusive transport, blood drainage, drug association with proteins and elimination in the tissue. Delivery results are assessed by drug penetration depth and drug distribution in space.

## 2. Material and methods

### 2.1. Model formulation

The mathematical model consists of a fluid transport model for ISF flow and a drug transport model for drug movement and accumulation in the anisotropic tissue. The transport of ISF is described by the mass equation and momentum conservation equation [18], as


(2.1) $$\nabla \cdot v=F_{BL}$$

(2.2) $$\frac{\partial \left(\rho v\right)}{\partial t}+\rho \left(v\cdot \nabla \right)v=-\nabla p_{ISF}+\mu \nabla^{2}v-\frac{\mu }{\kappa }v$$

where ρ is the ISF density, μ is the viscosity, v is the ISF velocity (IFV) and $p_{ISF}$ is the ISF pressure (IFP). The anisotropic tissue is treated as a porous medium in which the tissue permeability tensor is represented by κ, which is subject to the orientation of local axon bundles. K=κ/μ is the tissue hydraulic conductivity. The rate of fluid exchange between the blood vessels and the tissue ($F_{BL}$) is determined by Starling’s law [18], defined as


(2.3) $$F_{BL}=L_{BL}\frac{S_{BL}}{V_{TIS}}\left[p_{BL}-p_{ISF}-\sigma_{BL}\left(\pi_{BL}-\pi_{ISF}\right)\right]$$

where $L_{BL}$ is the hydraulic conductivity of the blood vessel walls. $S_{BL}/V_{TIS}$ is the area of the blood vessel wall per tissue volume, reflecting the blood vessel density. $\pi_{ISF}$ is the osmotic pressure of ISF, and $\pi_{BL}$ is that of the blood. $\sigma_{BL}$ refers to the osmotic reflection coefficient for proteins in the blood.

**Figure 1.**
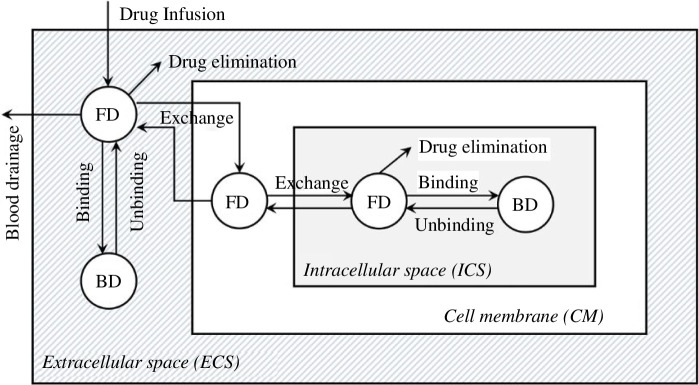
Schematical diagram of drug transport process in and between different tissue compartments. The striped area represents the local axon bundles in the tissue ECS, in which the transport of FDs relies on convection with the flow of ISF and concentration gradient-resulted diffusion. FDs can bind and unbind with proteins to form bound drugs (BDs) in the tissue ECS and ICS but are either bound with proteins or eliminated at the CM [19].

The brain tissue can be divided into three compartments, named the tissue intracellular space (ICS), cell membranes (CM) and extracellular space (ECS). The drug transport processes among different tissue compartments are schematically depicted in figure 1. The free drug (FD) travels in the tissue ECS by diffusion and convection.

Obeying the principle of conservation of mass, the concentration of drugs in their free forms ($C_{FD}$) and bound forms to proteins ($C_{BD}$) can be written as


(2.4) $$\begin{array}{c} C_{FD}=\epsilon_{ECS}C_{FD,ECS}+\epsilon_{CM}C_{FD,CM}+\epsilon_{ICS}C_{FD,ICS}\\ C_{BD}=\epsilon_{ECS}C_{BD,ECS}+\epsilon_{CM}C_{BD,CM}+\epsilon_{ICS}C_{BD,ICS} \end{array}$$

in which $\epsilon_{ECS}$, $\epsilon_{CM}$ and $\epsilon_{ICS}$ are the volume fraction of ECS, cell membrane and ICS, respectively. $C_{FD,ECS}$ and $C_{BD,ECS}$ are the concentrations of FDs and bound drugs (BDs) in the ECS. Similarly, $C_{FD,CM}$ and $C_{BD,CM}$ stands for FD and BD concentration on the cell membrane, respectively, and $C_{FD,ICS}$ and $C_{BD,ICS}$ are the concentrations of FDs and BDs in the ICS.

Drug delivery to tissues involves a series of processes, including diffusion in tissue ECS owing to the thermal motion of drug molecules, convective transport with ISF flow, blood drainage in ECS, binding with proteins in ICS and ECS, bioreactions and physical degradation in ICS and ECS, and cell uptake. Therefore, the FD concentration ($C_{FD}$) can be obtained [19] using


(2.5) $$\frac{\partial C_{FD}}{\partial t}=\nabla \cdot \left(\epsilon_{ECS}D_{FD,ECS}\nabla C_{FD,ECS}\right)-\nabla \cdot \left(\epsilon_{ECS}C_{FD,ECS}v\right)-\epsilon_{ECS}\left(k_{DR}+k_{EL}\right)C_{FD,ECS}-\epsilon_{ICS}k_{EL}C_{FD,ICS}-\frac{\partial C_{BD}}{\partial t}$$

where $D_{FD,ECS}$ is the diffusion tensor of FD in tissue ECS, depending on the orientation of local axon bundles. $k_{DR}$ is the rate of blood drainage. $k_{EL}$ is the rate of drug elimination by bioreactions and physical degradation. t is time.

Two assumptions are further introduced [19]. (i) The concentration of drug bound to proteins in the ECS and ICS is proportional to the corresponding FD concentration with a coefficient $K_{ECS}$ and $K_{ICS}$, respectively ($K_{ECS}=C_{BD,ECS}/C_{FD,ECS}$; $K_{ICS}=C_{BD,ICS}/C_{FD,ICS}$); (ii) the concentration of FDs in the ICS and on the cell membrane is proportional to the FD concentration in the ECS, with a coefficient of $P_{IE}$ and $P_{CE}$, respectively ($P_{IE}=C_{FD,ICS}/C_{FD,ECS}$; $P_{CE}=C_{FD,CM}/C_{FD,ECS}$). Therefore, equation (2.5) can be rewritten [9] as


(2.6) $$\frac{\partial C_{FD,ECS}}{\partial t}=\nabla \cdot \left(D_{FD,ECS}^{*}\nabla C_{FD,ECS}\right)-v^{*}\cdot \nabla C_{FD,ECS}-k_{E}^{*}C_{FD,ECS}$$

where the apparent diffusivity tensor of FD in tissue ECS is represented by $D_{FD,ECS}^{*}=\left(\epsilon_{ECS}/\omega \right)D_{FD,ECS}$. $v^{*}=\left(\epsilon_{ECS}/\omega \right)v$ is the apparent velocity of ISF flow. $k_{E}^{*}=k_{E}/\omega =\left[\epsilon_{ECS}k_{DR}+\left(\epsilon_{ECS}+\epsilon_{ICS}\right)k_{EL}+F_{BL}\right]/\omega$ is the drug’s apparent elimination rate. $\omega =\epsilon_{ECS}\left(1+K_{ECS}\right)+\epsilon_{ICS}P_{IE}\left(1+K_{ICS}\right)+\epsilon_{CM}P_{CE}$ is a parameter determined by the drug and tissue properties.

### 2.2. Model geometry and numerical implementation

**Figure 2.**
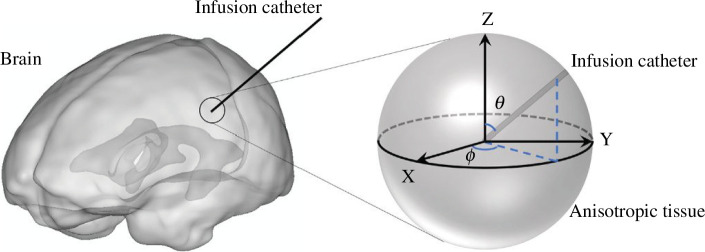
Model geometry. Drugs are infused into the brain tissue through the catheter, as shown in the left schematical diagram. The model geometry is given on the right, which focuses on a spherical volume around the infusion site. The track of axon bundles is in the X direction in this configuration. The angles between the catheter and the Z direction and the X direction are defined as θ and ϕ, respectively. Note that the left diagram is to present the studied process only. The position of the infusion catheter does indicate the location where the drugs are administrated.

The modelling of CED is performed in a three-dimensional spherical configuration as depicted in figure 2. The radius of the tissue domain (R) is 10 mm. Drugs are infused through a 21-gauge needle [20] with the infusion site positioned at the centre of the spherical domain. The infusion direction is defined by the two angles of θ and ϕ, which are the angles between the catheter and the Z direction and the X direction, respectively. The governing equations and boundary conditions captured in the proposed formulations are discretized using the finite elements framework and implemented in COMSOL Multiphysics [21]. The final computation mesh, which is generated to account for the strong local effects, consists of approximately $1.8\times 10^{6}$ elements based on a carefully conducted mesh-independence test; results are given in the electronic supplementary material. The finest elements with dimensions of 0.5 μm are applied to the infusion site and on the catheter wall.

### 2.3. Model parameters

Since drug delivery is commonly much more rapid compared with tissue growth, the tissue and drug properties are considered constant over time. To focus on the impact of infusion direction, the tissue properties are considered homogeneous and constant in the entire computational domain except for tissue anisotropy. Six common anti-brain tumour drugs are studied [22], including fluorouracil (5-FU), temozolomide (TMZ), carmustine (BCNU), cisplatin (CDDP), methotrexate (MTX) and paclitaxel (PTX). The model parameters and sources are given in tables 1 and 2, respectively. The infusant concentration ($C_{in}$) is set to be the drug solubility in water. The effective concentration ($C_{eff}$) is the drug concentration sufficient to cause 90% tumour cell death in *in vitro* experiments. The volume fraction of ECS ($\epsilon_{ECS}$) in the human brain white matter was reported to be 0.2 [24]. Since the water content of human brain white matter is approximately 0.75 [25], the volume fraction of ICS ($\epsilon_{ICS}$) is estimated to be 0.55, following the procedure applied in [26]. However, this may be an underestimation since organelles, which also occupy the ICS, can be excluded when measuring the water content. Owing to the lack of reporting of organelles volumes, the volume fraction of ICS ($\epsilon_{ICS}$) in the human brain white matter is averaged between the estimate and water content, set as 0.65 in this study. The justification for key model parameters is as follows.

**Table 1.** Model parameters of tissue property [18].

symbol	parameter	unit	value
ρ	ISF density	kg m^−3^	$1.0\times 10^{3}$
μ	ISF viscosity	Pa s	$7.8\times 10^{-4}$
$\pi_{BL}$	osmotic pressure of plasma	Pa	$3.4\times 10^{3}$
$\pi_{ISF}$	osmotic pressure of ISF	Pa	$7.4\times 10^{2}$
$p_{BL}$	blood pressure	Pa	$4.6\times 10^{3}$
$S_{BL}/V_{TIS}$	surface area of blood vessel wall per tissue volume	m^−1^	$7.0\times 10^{3}$
$\sigma_{BL}$	osmotic reflection coefficient for proteins in the blood	-	$9.1\times 10^{-1}$
$L_{BL}$	hydraulic conductivity of the wall of blood vessels	m Pa^−1^ s^−1^	$1.4\times 10^{-13}$
$\kappa_{TIS}$	averaged hydraulic permeability of tissues	$m^{2}$m^2^	$7.1\times 10^{-15}$

**Table 2.** Model parameters of drug properties [18,23].

symbol	parameter	unit	fluorouracil	temozolomide	carmustine	cisplatin	methotrexate	paclitaxel
MW	molecular weight	Da	130.08	194.15	300.01	454.44	543.52	853.91
$P_{IE}$	ICS-ECS partition coefficient	-	1.0	1.0	1.0	1.0	1.0	1.0
$P_{CE}$	CM-ECS partition coefficient	-	0.1	0.015	10.3	0.006	0.01	3162.3
$K_{ECS}$ *,* $K_{ICS}$	drug bound to protein coefficient	-	0.1	0.18	5.0	1.0	0.7	5.1
$D_{FD,ECS}$	averaged diffusivity of FD	m^2^ s^−1^	$1.2\times 10^{-9}$	$3.4\times 10^{-10}$	$1.5\times 10^{-9}$	$2.5\times 10^{-10}$	$5.3\times 10^{-10}$	$9.0\times 10^{-10}$
$k_{BD}$	drug blood drainage rate	$s^{-1}$	$1.8\times 10^{-2}$	$1.1\times 10^{-4}$	$1.4\times 10^{-2}$	$2.9\times 10^{-2}$	$2.8\times 10^{-4}$	$1.4\times 10^{-4}$
$k_{EL}$	drug physical degradation rate	$s^{-1}$	$5.6\times 10^{-4}$	$1.1\times 10^{-4}$	$1.1\times 10^{-4}$	$7.3\times 10^{-4}$	$1.5\times 10^{-4}$	$6.8\times 10^{-7}$
$C_{in}$	infusant concentration	M	$7.7\times 10^{-3}$	$1.6\times 10^{-2}$	$1.9\times 10^{-2}$	$3.3\times 10^{-3}$	$3.7\times 10^{-4}$	$7.0\times 10^{-6}$
$C_{eff}$	effective concentration, ${LD}_{90}$,	M	$2.0\times 10^{-6}$	$3.9\times 10^{-5}$	$1.5\times 10^{-5}$	$2.0\times 10^{-5}$	$5.9\times 10^{-5}$	$8.9\times 10^{-7}$

#### 2.3.1. Infusion rate

The infusion rate usually ranges from 0.5 to 10 μl min^−1^ to avoid significant tissue deformation and possible damage to the soft brain tissue [27,28]. Previous studies also showed that the catheter can be left in the brain for long-term infusion when the infusion rate is less than 5.0 μl min^−1^ [5]. Hence, the rate of 1.0 μl min^−1^ is selected.

#### 2.3.2. Anisotropic characteristics

The tissue anisotropy is usually presented as a tensor which has three components, including an axial principal value and two similar radial principal values; they are along and perpendicular to the local axon bundles, respectively. Determined by the arrangement of local nerve fibres, the differences between the axial and radial principal values vary greatly across the whole brain [29]. For instance, these three components are comparable in the putamen, whereas the axial one is about 10 times higher in the region close to the corpus callosum [14]. Therefore, in this study, the axial principle value is set to be 9.0 times the radial principle value as a representative situation. Tissue anisotropy is reflected by the transport properties of tissue permeability (κ) [30,31] and drug diffusivity ($D_{FD,ECS}$) in the drug delivery model [14,32]. Since they share the same changing pattern with the local nerve fibres [6], the factor of 9.0 is applied to scale the base values of these two model parameters in tables 1 and 2 to obtain their components in the axial direction (the X direction) and the two radial directions (the Y and Z directions), respectively, as given in table 3.

**Table 3.** Tensor of anisotropic tissue permeability and drug diffusion coefficient.

	tissue permeability ($m^{2}$)	drug diffusion coefficient (m^2^ s^−1^)					
		fluorouracil	temozolomide	carmustine	cisplatin	methotrexate	paclitaxel
along axons (X direction)	$7.06\times 10^{-15}$	$1.19\times 10^{-9}$	$3.38\times 10^{-10}$	$1.49\times 10^{-9}$	$2.48\times 10^{-10}$	$5.27\times 10^{-10}$	$8.94\times 10^{-10}$
perpendicular to axons (Y and Z directions)	$7.84\times 10^{-16}$	$1.33\times 10^{-10}$	$3.75\times 10^{-11}$	$1.66\times 10^{-10}$	$2.76\times 10^{-11}$	$5.85\times 10^{-11}$	$9.94\times 10^{-11}$

#### 2.3.3. Infusion direction

The placement of the infusion catheter is controlled by the two angles of θ and ϕ simultaneously. These two angles vary independently in the range of 0°–90°, with a change interval of $10^{o}$ in the following simulations. Specifically, when θ=90° and ϕ=0°, the infusion catheter aligns with the axon track in the X direction. The infusion catheter is located in the Y direction, which is perpendicular to the axon track when θ=90° and ϕ=90°. No matter the value of ϕ, the infusion catheter is in the Z direction perpendicular to the axon tracks when θ=0°.

### 2.4. Boundary conditions

The gauge pressure and drug flux at the outer surface of the anisotropic tissue is zero. The wall of the infusion catheter is rigid with zero drug flux. A constant flow rate and infusant concentration in table 2 are imposed at the infusion site for each drug, respectively.

Drugs in clinical trials typically require infusion for 3 to 5 days [27], which is sufficient to allow the infusion to dynamically equilibrate with the fluid and drug loss to the surrounding environment and downstream tissues [18,33]. Therefore, this study focuses on the delivery outcomes in this quasi-steady state.

### 2.5. Qualification index

Drug delivery results under different conditions are examined using the following qualification indicators.

#### 2.5.1. Effective penetration depth

Drug penetration from the infusion site is non-uniform in the brain tissue, subject to the local axons. The effective penetration depth, $H_{eff}$, is applied to study the drug’s effective transport in each direction, as


(2.7) $$\begin{array}{ccc} H_{X,eff,i}=\left|x-x_{0}\right|; & H_{Y,eff,i}=\left|y-y_{0}\right|; & H_{Z,eff,i}=\left|z-z_{0}\right| \end{array}$$

in which $\left(x_{0},y_{0},z_{0}\right)$ marks the infusion site. (x,y,z) defines the location where the FD concentration in tissue ECS ($C_{FD,ECS}$) falls to the drug’s effective concentration ($C_{eff}$). The subscript i can be either ant or post, referring to the position in front of or behind the infusion catheter tip, respectively.

#### 2.5.2. Penetration asymmetry

The dimensionless number, penetration asymmetry (AS), is introduced to compare drug penetration depth in front of and behind the infusion catheter tip in a certain direction, i.e. along or perpendicular to the local nerve fibres. The penetration AS in each direction can be calculated by


(2.8) $$\begin{array}{ccc} {AS}_{X}=H_{X,eff,ant}/H_{X,eff,post}; & {AS}_{Y}=H_{Y,eff,ant}/H_{Y,eff,post}; & {AS}_{Z}=H_{Z,eff,ant}/H_{Z,eff,post} \end{array}$$

A higher value of AS indicates drug penetration is more effective in front of the infusion catheter in the examined direction.

#### 2.5.3. Fractional anisotropy

Fractional anisotropy (FA) is a dimensionless number that evaluates the degree of AS of a drug spatial distribution volume, defined as


(2.9) $$FA=\sqrt{\frac{{\left(H_{X,eff}-H_{Y,eff}\right)}^{2}+{\left(H_{Y,eff}-H_{Z,eff}\right)}^{2}+{\left(H_{X,eff}-H_{Z,eff}\right)}^{2}}{2\left(H_{X,eff}^{2}+H_{Y,eff}^{2}+H_{Z,eff}^{2}\right)}}$$

where $H_{X,eff}=H_{X,eff,ant}+H_{X,eff,post}$ is the total effective penetration depth in the X direction, considering the penetration both in front of and behind the infusion catheter tip. Similarly, the total effective penetration depth in the Y and Z directions is defined as $H_{Y,eff}=H_{Y,eff,ant}+H_{Y,eff,post}$ and $H_{Z,eff}=H_{Z,eff,ant}+H_{Z,eff,post}$, respectively. The value of FA varies between 0 and 1, with a higher value presenting a more anisotropic drug distribution in space.

#### 2.5.4. Directional anisotropy

Drug penetration can be different between the directions along and perpendicular to the local nerve fibres. In order to compare in which direction the drug can penetrate deeper, the dimensionless number, directional anisotropy (DA), is used. It is defined as


(2.10) $$\begin{array}{ccc} {DA}_{X}=\frac{H_{X,eff}}{2R_{equiv}}; & {DA}_{Y}=\frac{H_{Y,eff}}{2R_{equiv}}; & {DA}_{Z}=\frac{H_{Z,eff}}{2R_{equiv}} \end{array}$$

The $R_{equiv}$ is the equivalent radius of drug effective distribution volume, in which the local FD concentration is greater than $C_{eff}$. Higher values indicate that the drug is more effective in penetrating in the corresponding direction.

#### 2.5.5. Enhanced flow coverage ($V_{En-ISF,(\theta ,\varphi )}$)

CED improves drug transport by accelerating ISF flow. Therefore, it is necessary to determine the volume of the region where the flow is enhanced. This volume, $V_{En-ISF\;\left(\theta ,\varphi \right)}$, at a specific infusion (θ,φ) direction can be calculated using


(2.11) $$V_{En-ISF,(\theta ,\varphi )}=\sum V_{j}\left(v_{ISF,j}\geq TH\right)$$

where $V_{j}$ is the local tissue volume, and $v_{ISF,j}$ is the local IFV magnitude. TH is a threshold to evaluate ISF flow enhancement. It is set as 30%, 10%, 5% and 1% of the infusion velocity ($u_{in}$) in this study.

## 3. Results

### 3.1. Model validation

**Figure 3.**
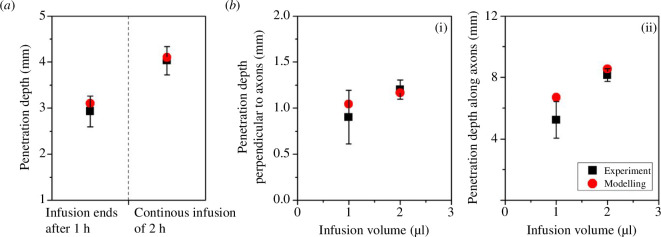
Model validation. The modelling predictions are compared with the reported experimental measurements [34,35]. (*a*) Infusion into isotropic material. The 0.5 μl min^−1^ infusion is through a 30 gauge needle. The hydraulic conductivity of agarose gel is $1.0\times 10^{-14}\;m^{4}\;N^{-1}\;s^{-1}$, and the diffusivity of trypan blue in agarose gel is $3.0\times 10^{-10}\;m^{2}\;s^{-1}$ [34]. (*b*) Infusion into anisotropic brain white matter. The 0.1 μl min^−1^ infusion is through a 33 gauge needle. The permeability of rat brain tissue along and perpendicular to axons is $4.49\times 10^{-15}m^{2}$ and $4.49\times 10^{-16}m^{2}$, respectively [35]. The diffusivity of albumin is $2.29\times 10^{-11}\;m^{2}\;s^{-1}$ and $1.34\times 10^{-11}\;m^{2}\;s^{-1}$ in these two directions, respectively [35]. The subplots (i) and (ii) show the penetration depths in the directions vertically and along the axons, respectively. The experimental results and model parameters applied in these two validation studies, including infusion parameters, drug properties and tissue properties, are extracted from [34,35], respectively.

The predictive accuracy of the mathematical model needs to be verified before simulating drug delivery. The model is first validated by predicting the delivery into isotropic materials, with results presented in figure 3*a*. Specifically, trypan blue is infused at the rate of 0.5 μl min^−1^ into the agarose gel in the first 1 h, followed by (left) 1 h diffusion only and (right) 1 h continuous infusion, respectively. The comparisons show the modelling predictions well agree with experimental measurements reported in [34]. Furthermore, the model is applied to simulate the delivery of albumin in the anisotropic materials. To be consistent with the experiments, 1 and 2 μl infusate are administrated in the white matter at the rate of 0.1 μl min^−1^. As shown in figure 3*b*, the predicted penetration depths along and perpendicular to the axons are in good agreement with the experimental results [35]. Therefore, the model is validated and applied in the subsequent simulations.

### 3.2. Drug delivery under baseline condition

**Figure 4.**
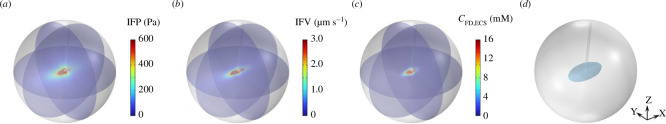
Delivery outcomes of infusing drugs into the anisotropic tissue (θ=45°; ϕ=45°). The spatial distribution of IFP (*a*), IFV (*b*) and TMZ concentration (*c*). The spatial distribution in which the concentration of TMZ is greater than its $C_{eff}$, marked in cyan in (*d*).

Delivery of the six drugs into the anisotropic tissue is first studied under the baseline condition with the infusion catheter positioned at $\theta =45^{\circ }$ and $\phi =45^{\circ }$. Drug movement and deposition in CED heavily depend on the hydraulic environment in the tissue. The transport of ISF and drug is simulated by solving the governing equations in the entire computational domain, subject to the tissue properties and drug properties given in tables 1–3. The IFP and IFV are shown in figure 4*a* and *b*, respectively. Results demonstrate the effectiveness of infusion in raising IFP, thereby generating a sharp pressure gradient from the infusion site to distal tissue. Consequently, the ISF flow can be successfully accelerated, as displayed in figure 4*b*. A comparison between different directions further shows that ISF flows more readily along the axon bundles due to higher tissue permeability in this direction. From a quantitative analysis, only in a region with a volume of 10.4 mm^3^ does the IFV exceed 1% of the velocity at the injection site; its equivalent radius is 1.35 mm, which is much smaller than the dimension of the computational domain. This finding indicates that the flow velocity drops fast as the distance from the catheter tip increases, resulting in the enhancement of ISF flow being limited to a small region around the infusion site.

The spatial distribution of TMZ concentration in figure 4*c* presents a similar pattern to the ISF flow. The concentration achieves its peak at the infusion site, where the drug is administrated, and decreases with increasing distance from the catheter tip. This decrease in the X direction, which aligns with the axon bundles, is slower than in the Y and Z directions, which are perpendicular to the axons. This is because, on one hand, the ISF flows more rapidly in the X direction, as shown in figure 4*b*, leading to faster convective movement of the drug. On the other hand, the drug diffusivity is also higher along the direction of the axons [13,14], making drug transport by thermal motion more efficient in that direction. The volume of TMZ with effective therapeutic concentration is shown in figure 4*d*. The drug can travel into deeper tissue along the axon bundles, although the infusion catheter is not fully aligned in this direction. It is worth noting that the drug also accumulates at the back of the infusion catheter, making the effective distribution approximate an ellipsoid. The other five drugs show similar distribution patterns, which are not presented here due to space limitations. The distribution volume anisotropy is comparable among the different drugs, with their FA located in the range of 0.49±0.01.

**Figure 5.**
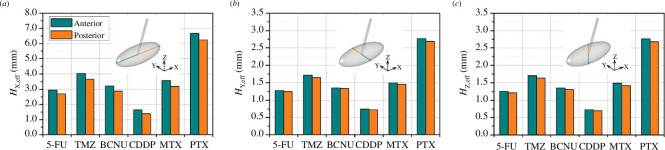
Comparison of effective penetration depth ($H_{eff}$) between different drugs under the baseline delivery condition of θ=45° and ϕ=45°. The effective penetration depth in the X, Y and Z directions are shown in (*a*), (*b*) and (*c*), respectively. The penetration depth in front of and behind the infusion catheter tip is marked in dark cyan and orange, respectively.

The effective penetration depth ($H_{eff}$) of each drug in the directions along and perpendicular to the nerve fibres are compared in figure 5. The drug diffusion coefficient in each direction is given in table 3. Results show that the effective penetration depth of all drugs presents a similar trend in all directions. PTX has the deepest penetration, followed by TMZ, MTX, BCNU and 5-FU; whereas, CDDP has the most limited travel distance into the tissue. The comparison across different directions shows that all drugs can penetrate much deeper along the axon bundles. In contrast, the penetration depths in the other two radial directions are comparable but relatively short. This can be attributed to the anisotropic nature of the tissue. Moreover, in the same direction, all the drugs can travel slightly farther in front of the infusion catheter than at the back of the catheter. This difference is slightly pronounced in the direction along the axon bundles.

### 3.3. Impact of infusion direction on ISF flow

**Figure 6.**
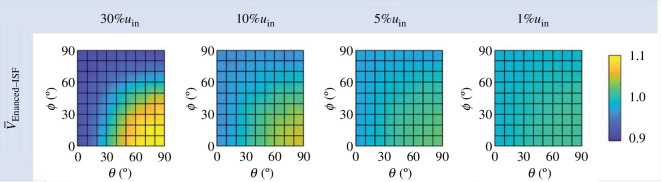
Effect of infusion direction on ISF flow. It is evaluated in terms of the normalized enhanced flow coverage (${\overset{-}{V}}_{En-ISF,\left(\theta ,\varphi \right)}$), calculated by normalizing $V_{En-ISF,\left(\theta ,\varphi \right)}$ at different infusion directions by the value at the baseline delivery condition of θ=45° and ϕ=45°.

Figure 6 shows the calculated enhanced flow coverage ($V_{En-ISF,(\theta ,\varphi )}$) using thresholds (TH) of 30%, 10%, 5% and 1% of the infusion velocity ($u_{in}$). Note that to cross-compare this qualification index obtained at these different thresholds, the enhanced flow coverages are further normalized by the corresponding values obtained under the baseline delivery condition, $V_{En-ISF,(\theta =45{}^{\circ},\varphi =45{}^{\circ})}$, which are 0.065, 0.328, 0.923 and 10.399 mm^3^, respectively.

The simulation results in figure 6 show that the volume of the region with enhanced ISF can be enlarged as the infusion catheter gets aligned with the axon track (θ=90° and ϕ=0°). On the contrary, this volume reaches the minimum when the infusion catheter is perpendicular to the axon bundles. Furthermore, since IFV decreases when the flow penetrates deep into the tissue, as shown in figure 4, a lower threshold (TH) indicates a longer distance from the infusion site. Therefore, cross-comparisons between the results obtained at different thresholds denote that the impact of infusion direction reduces with increasing distance from the infusion site. Specifically, the enhanced flow coverage obtained at 1% of $u_{in}$ remains relatively constant regardless of the infusion direction. Further analysis shows that adjusting the infusion direction has limited influence on the overall IFV in the entire domain, largely due to its impact on the enhanced ISF being restricted in the region around the infusion site, as shown in figure 4*a,b*.

### 3.4. Impact of infusion direction on drug distribution

**Figure 7.**
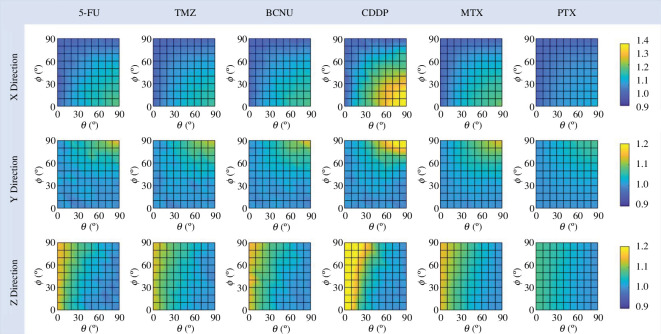
Comparison of drug penetration AS with infusion catheters in different directions.

Figure 7 presents the impact of infusion direction on the penetration AS of each drug. This indicator evaluates the difference between penetration at the front and back of the catheter, as defined in equation (2.8). The effective penetration of CDDP shows the most significant response to infusion direction. In contrast, changes in the penetration AS of PTX are relatively small. Regardless of drug type, this difference in a particular direction becomes larger when the infusion catheter moves towards that direction. Specifically, raising θ or reducing ϕ can effectively increase the degree of penetration AS in the X direction, which is along the axon bundles. The penetration in the Y direction gets more asymmetric when θ or ϕ increases, whereas the most asymmetric distribution in the Z direction occurs at θ=0°. A quantitative comparison further shows that the increase in AS can be mainly attributed to the reduction in penetration at the back of the catheter.

**Figure 8.**
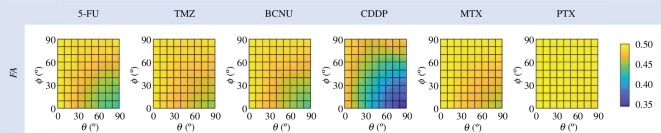
Effect of infusion direction on FA of effective drug distribution.

The dependence of the FA of each drug on the infusion direction is shown in figure 8. The distributions of all types of drugs are anisotropic in space subject to the nature of the tissue. However, this distribution becomes more isotropic with the increased θ or decreased ϕ. The lowest degree of FA occurs when the infusion catheter is placed along the axon track (θ=90° and ϕ=0°). Cross-comparison between different drugs further shows that the FA of CDDP responds most markedly to changes in infusion direction, while the distribution of PTX remains more anisotropic no matter how infusion direction changes. The different sensitivities are primarily because of differences in the drug’s penetration ability. Drug transport depends on the mechanisms of concentration gradient-resulted diffusion and convection with the flow of ISF. Although infusion direction can effectively alter the ISF flow, its influence is confined within a limited region around the infusion site, as shown in figures 4 and 6. Therefore, only the drugs with a short penetration depth react to the change in infusion direction significantly, such as CDDP and 5-FU. On the contrary, for those drugs which can penetrate deep in the tissue, such as PTX, their transport in the distal region far from the infusion site is more determined by diffusion, which is independent of the ISF flow. So their FA is less affected by infusion direction.

**Figure 9.**
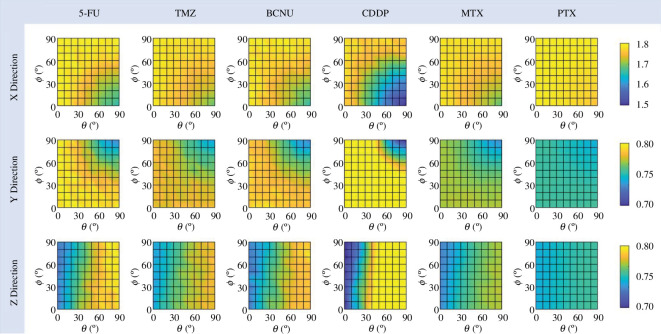
Effect of infusion direction on the degree of DA.

Figure 9 compares the DA of each drug in different infusion directions. The results show that this degree in a given direction gradually decreases as the infusion catheter moves more in that direction. Specifically, the anisotropy in the X direction becomes most insignificant when the infusion direction is parallel to the orientation of the axon bundles; similar trends can be found in the Y and Z directions. A follow-up quantitative analysis identifies the reduction in the penetration depth at the back of the catheter as the major reason. Comparing different drugs, the degree of anisotropy of CDDP in each direction has a more marked response to the changes in the placement of the infusion catheter. Differently, the drug distribution of PTX is less responsive.

## 4. Discussion

CED is developed to directly infuse drugs into the brain tissue to overcome the BBB and improve drug convective transport with ISF flow. Simulations in this study demonstrate that this infusion can successfully accelerate ISF flow and provide a friendly hydraulic environment for drug transport. However, this improvement strongly depends on the orientation of local axon bundles, exhibiting a high degree of anisotropy. More importantly, the increased velocity rapidly drops within a short distance from the catheter tip, limiting this improvement within a small volume around the infusion site. Drug transport in the ECS of brain tissue primarily depends on diffusion and convection, while the latter could be dominant. Since both processes are anisotropic and more efficient along the axons than perpendicular to the axons, the spatial distribution of drugs in three-dimensional exhibits an elliptical shape.

This highly non-uniform enhancement of ISF flow induced by CED is one of the main reasons for the large differences between drugs in response to changes in infusion direction. For drugs with shorter penetration distances, their distribution largely depends on the ISF flow at the infusion site, which is significantly affected by the infusion direction, as shown in figure 6. In contrast, the transport of drugs with greater penetration ability in deep tissues relies more on diffusion, a process independent of the convection with the flow and thus less influenced by infusion direction. As shown in figure 5, CDDP presents the shortest travel distance from the infusion site, followed by 5-FU and BCNU; TMZ and MTX show improved penetration, while PTX can be delivered to the deepest tissue compared to other drugs. This order is consistent with the findings from figure 8, which shows that CDDP’s distribution has the most significant response to the change in infusion direction, whereas the distribution of PTX is less sensitive to this change.

Within the same ISF flow field, drug penetration depth can be evaluated in terms of the Thiele modulus (Φ) [18], defined as


(4.1) $$\Phi =R\sqrt{k_{E}^{*}/D_{FD,ECS}^{*}}$$

Thiele modulus compares the contributions of elimination and diffusion in determining drug transport under the same hydraulic environment in the tissue, with a higher value indicating a more important role of elimination. Thiele modulus of the six drugs is summarized in table 4, presenting the same order as that in figure 4 according to penetration depth. The lowest Thiele modulus of PTX implies its transport is less determined by elimination so that more drug is maintained in the tissue to travel into deep tissue. On the contrary, most CDDP is eliminated before travelling into deep tissue, exhibiting the most limited penetration compared to other drugs.

**Table 4.** Thiele modulus of different drugs.

drug	5-FU	TMZ	BCNU	CDDP	MTX	PTX
Thiele modulus	41.22	13.15	31.07	113.34	13.23	4.13
Change in FA	14.86%	9.17%	12.28%	30.06%	9.40%	3.29%

The range in variation of FA of each drug compared to its FA obtained under the baseline delivery condition is also given in table 4. The drugs appear in the same order as those arranged by Thiele modulus. This finding highlights the importance of drug selection and the development of specific delivery regimens for different drugs. For drugs that can penetrate deep into anisotropic tissues, the distribution is less affected by infusion direction because drug transport in areas away from the infusion site is still diffusion dominated. This insensitivity to infusion direction allows greater flexibility in catheter insertion and placement. On the contrary, infusing short-penetrating drugs along the axon bundles can reduce the difference in penetration depth between different directions, thereby making the drug distribution relatively more isotropic. Further quantitative analyses show the averaged drug concentration and overall effective distribution volume are less affected by infusion direction. However, the directional responses of drug spatial distribution also suggest that the infusion direction needs to be well planned and controlled. For lesions with obvious directional distribution along the orientation of nerve fibres, placing the infusion catheter in a direction perpendicular to the nerve fibres can better achieve localized drug delivery and reduce the impact on surrounding tissues. In contrast, infusing drugs along the direction of axon bundles can relatively improve the uniformity of drug distribution. This precise control of infusion direction can be achieved with a bevel-tip steerable catheter consisting of four interlocked segments [36]. This wasp-inspired flexible catheter can instantly adjust the direction of insertion by controlling the relative movement between its four segments [37]. With the further support of a front-end catheter path planner and a visual navigation system [38], it can follow a pre-designed, curved pathway towards the selected infusion site [39].

Drug delivery outcomes through CED greatly depend on the delivery protocol and clinical settings, particularly the placement of infusion catheters. It has been reported that combining catheter movement with constant pressure infusion can significantly increase drug dispersed volume, while infusion with retraction is conducive to uniform drug distribution [40]. Attention also needs to be given to the selection of infusion sites since the original ISF flow at and around the site can play a role in determining drug accumulation and distribution [12]. This study is focused on the impact of infusion direction, however, modelling studies can be carried out in future to consider different clinical settings to optimize the CED delivery protocol for improved outcomes and efficacy.

The IFP and IFV in the brain tissue are predicted to be 19.48 Pa and $4.63\times 10^{-8}\;m\;s^{-1}$, respectively, upon CED infusion, and 2.19 Pa and $5.26\times 10^{-9}\;m\;s^{-1}$ without CED infusion. These predictions are located in the ranges of 0–1333 Pa and $8.3\times 10^{-10}\;∼\;4.17\times 10^{-6}\;m\;s^{-1}$, respectively, reported in [41]. Model validation is performed in this study and also reported in the literature. For instance, the IFP in solid tumours and the holding tissues were predicted as 1500 and 40 Pa [42], respectively, which were located in the ranges of 587–4200 and −400 to 800 Pa measured in experiments [43]. A drug delivery model was used to predict the distribution volume of albumin when infused into agarose gels. The coefficient of determination was calculated as 0.8 compared with the experimentally measured data [44]. However, it is still worth mentioning that modelling predicted drug delivery outcomes are usually qualitative. These predictions can only be used to determine the role of influencing factors through cross-comparisons.

This modelling study also involves some assumptions and limitations. (i) Uniform anisotropic properties are mapped in the entire computational domain that represents a small region around the infusion site. This is mainly because the change in tissue anisotropy in the local tissues is more gradual as a result of the continuous axon bundles. Notably, the degree of anisotropy can vary greatly across the entire brain white matter. This limitation can be overcome by using DTI data from which the anisotropic characteristics of the entire brain can be derived pixel-wise [14]. (ii) Backflow is a major limitation of CED. Pushing brain tissue in the opposite direction against the outer surface of the catheter, CED infusion can lead to a teardrop-shaped space surrounding the catheter tip. Drugs accumulating in this space have the potential to travel along the catheter tack to normal brain tissues. Consequently, backflow can not only reduce drug availability at the lesion site but also cause undesired side effects. However, one should also note that backflow can increase the surface area through which drugs can enter the brain tissues. This enlargement may enhance the interaction of CED with local nerve fibres, particularly those behind the catheter, thereby affecting delivery outcomes. The formation of backflow is determined by several factors [45,46], including infusion rate, brain interstitial pressure, tissue deformation and damage, catheter insertion and implementation and oedema. Moreover, increased pressure at the infusion site can also deform the local tissues and as such alter tissue permeability [47]. Since these infusion-introduced changes in tissue properties are positively correlated with the infusion rate and are usually orders lower compared with the length scale of drug transport, tissue deformation is not considered when drugs are infused at a low rate [48]. However, the drug concentration may be underestimated when the infusion pressure or infusion rate is high [47]. This assumption can be relaxed by using an advanced tissue mechanics model, such as the hyperelastic model [49] or hyper-viscoelastic model [50], and semi-empirical formulas of tissue permeability [51] to consider the fluid–tissue interactions. Furthermore, advanced catheters have also been developed to reduce backflow [52]. Future modelling studies can explore the CED drug infusion using these catheters with improved designs. (iii) The BBB is usually considered intact in the modelling of brain drug delivery. However, it was reported that the blood vessels can be leaky in high-grade brain tumours and metastatic brain tumours [53]. Moreover, brain tissue properties, such as blood vessel density, vascular permeability, tissue permeability, extracellular matrix density and blood perfusion rate, can be highly heterogeneous, particularly in brain solid tumours. Their importance to delivery outcomes may also differ from each other [54]. In order to focus on infusion direction, the impact of these heterogeneous properties is not considered in the current study. To relax this assumption, such tissue properties can be obtained from patient medical images with different modalities, including MRI, computed tomography, PET and ultrasound [55]. Using these properties that can reflect a more realistic *in vivo* microenvironment is able to improve the accuracy of modelling predictions of drug delivery outcomes. Furthermore, tumour vasculature, including the morphological features [56] and vessel wall structures [57], can become abnormal with the tumour growth. This abnormality is considered in terms of the blood vessel density and vascular permeability in the drug delivery models at the tissue scale [54]. Although these two parameters can also be extracted from medical images [55], the resolution is limited by the image pixel size which is usually in millimetres. The microscale models, in which the blood vessels are simulated explicitly [58,59], can be applied to provide drug delivery predictions with higher resolution. (iv) Notably, the modelling of drug delivery requires a large number of model parameters. Subject to data availability, representative values from *in vitro* and *in vivo* experiments using different animals are adopted, making modelling predictions qualitative. For more detailed quantitative analyses, patient-specific information would be needed. This information can be obtained from biopsy and patient medical imaging data.

## 5. Conclusions

The effect of infusion direction on the CED of six drugs into anisotropic tissue has been studied using a validated mathematical model. The simulation results denote the effectiveness of infusion in accelerating the ISF flow and thence enhancing the drug transport. This improvement is highly anisotropic subject to the orientation of local axon bundles and is limited in a small region around the infusion site. Moving the infusion catheter in a direction parallel or perpendicular to the axon can effectively reduce the contribution of drug penetration in that direction to the overall distribution anisotropy. This is mainly caused by the reduction of drug penetration at the back of the infusion catheter relative to the front. Cross-comparisons reveal great differences in the sensitivity of delivery outcomes of different drugs to infusion direction. The distribution of CDDP, 5-FU and BCNU becomes more isotropic when the infusion catheter is more aligned with the local axon bundles. However, the distributions of PTX, MTX and TMZ are less affected by the placement of the infusion catheter. Since catheter placement is a factor that can be precisely controlled in practice, these results can aid in planning treatments using CED, particularly catheter insertion and placement.

## Data Availability

Supplementary material is available online at [60].
